# Microanatomical and Histological Features in the Long Bones of Mosasaurine Mosasaurs (Reptilia, Squamata) – Implications for Aquatic Adaptation and Growth Rates

**DOI:** 10.1371/journal.pone.0076741

**Published:** 2013-10-16

**Authors:** Alexandra Houssaye, Johan Lindgren, Rodrigo Pellegrini, Andrew H. Lee, Damien Germain, Michael J. Polcyn

**Affiliations:** 1 Steinmann Institut für Geologie, Paläontologie und Mineralogie, Universität Bonn, Bonn, Germany; 2 Department of Geology, Lund University, Lund, Sweden; 3 New Jersey State Museum, Trenton, New Jersey, United States of America; 4 Department of Anatomy, Midwestern University, Glendale, Arizona, United States of America; 5 UMR7207 CNRS-MNHN-UPMC, Département Histoire de la Terre, Muséum National d’Histoire Naturelle, Paris, France; 6 Huffington Department of Earth Sciences, Southern Methodist University, Dallas, Texas, United States of America; Raymond M. Alf Museum of Paleontology, United States of America

## Abstract

**Background:**

During their evolution in the Late Cretaceous, mosasauroids attained a worldwide distribution, accompanied by a marked increase in body size and open ocean adaptations. This transition from land-dwellers to highly marine-adapted forms is readily apparent not only at the gross anatomic level but also in their inner bone architecture, which underwent profound modifications.

**Methodology/Principal Findings:**

The present contribution describes, both qualitatively and quantitatively, the internal organization (microanatomy) and tissue types and characteristics (histology) of propodial and epipodial bones in one lineage of mosasauroids; i.e., the subfamily Mosasaurinae. By using microanatomical and histological data from limb bones in combination with recently acquired knowledge on the inner structure of ribs and vertebrae, and through comparisons with extant squamates and semi-aquatic to fully marine amniotes, we infer possible implications on mosasaurine evolution, aquatic adaptation, growth rates, and basal metabolic rates. Notably, we observe the occurrence of an unusual type of parallel-fibered bone, with large and randomly shaped osteocyte lacunae (otherwise typical of fibrous bone) and particular microanatomical features in *Dallasaurus*, which displays, rather than a spongious inner organization, bone mass increase in its humeri and a tubular organization in its femora and ribs.

**Conclusions/Significance:**

The dominance of an unusual type of parallel-fibered bone suggests growth rates and, by extension, basal metabolic rates intermediate between that of the extant leatherback turtle, *Dermochelys*, and those suggested for plesiosaur and ichthyosaur reptiles. Moreover, the microanatomical features of the relatively primitive genus *Dallasaurus* differ from those of more derived mosasaurines, indicating an intermediate stage of adaptation for a marine existence. The more complete image of the various microanatomical trends observed in mosasaurine skeletal elements supports the evolutionary convergence between this lineage of secondarily aquatically adapted squamates and cetaceans in the ecological transition from a coastal to a pelagic lifestyle.

## Introduction

Mosasauroidea includes medium-sized to giant lizards that evolved in the oceans of the Late Cretaceous between 98 and 66 Ma ago (e.g. [1], [2]). During their evolution, mosasauroids acquired a worldwide distribution, accompanied by a marked increase in body size (some forms exceed 15 meters in length) and open-ocean adaptations [3], [4], [5]. Three general morphotypes have been recognized [6], [7] that seemingly illustrate progressive steps in the adaptation of mosasauroids to open-ocean habitats, as well as increasing abilities for more energy-efficient swimming. These morphotypes include: (1) rather small forms (typically <2 meters long) that display terrestrial-like (plesiopedal) limbs and a typical squamate pelvic girdle (i.e., a plesiopelvic anatomy); (2) taxa (from 3 to 6 meters long) possessing plesiopedal limbs but where the pelvic girdle has been transformed so that there is no sacral contact with the ilium (i.e., a hydropelvic anatomy); and (3) forms (from 4 to 15 meters long) that have both paddle-like (hydropedal) limbs and a hydropelvic anatomy.

Certain aspects of the biomechanics and physiology of mosasauroids are reflected in their postcranial skeleton. At the microanatomical level (i.e., in the bone inner organization) are notably observed specializations related to the mechanical constraints of locomotion and to the hydrostatic or hydrodynamic control of buoyancy (i.e., in order to remain submerged) and body trim (i.e., to retain a horizontal body orientation) in water. For instance, plesiopelvic forms are known to display bone mass increase (*sensu* [8]) in at least their vertebrae and ribs [9], [10] and, conversely, hydropelvic forms have a spongious inner bone organization [9], [11], [12]. Specializations at the histological (tissue) level also occur; the prevalence of highly vascularized parallel-fibered bone has been associated with relatively high growth rates and maintenance of relatively high core body temperatures suggesting elevated basal metabolic rates compared to extant squamates [12], [13].

Osteohistological studies of mosasauroids have hitherto been limited to ribs [11], [12], [14], [15] and vertebrae [9], [10], [12], [16], [17], whereas mosasauroid long bones have been analyzed solely from a skeletochronological perspective [18]. This lack of information precludes meaningful histological and microanatomical comparisons with other groups of obligate marine reptiles for which only long bone data are available [13]. Accordingly, in the present contribution, we describe, both qualitatively and quantitatively, microanatomical and histological specializations observed in mid-diaphyseal sections of pro- and epipodial bones (see [19]) in one lineage of mosasauroids; i.e., the subfamily Mosasaurinae, and compare these features with those observed in extant squamates and extant and extinct semi-aquatic to fully marine amniotes. We conclude with a discussion on the implications for mosasaurine evolution, including increased growth rates and elevated basal metabolic rates.

## Materials and Methods

The material selected for osteohistological analysis comprises pro- and epi-podial bones representing six genera of mosasaurine mosasaurs (i.e., the plesiopedal and hydropelvic *Dallasaurus*, and the hydropedal and hydropelvic *Clidastes*, *Globidens*, *Mosasaurus*, *Plotosaurus*, and *Prognathodon*) that collectively record approximately 26 million years of evolution in a single mosasauroid lineage (Table 1). We chose humeri whenever possible because propodials have a stronger ecological signal than epipodials [20], [21] and because femora are rare in collections. Comparative material includes sectioned humeri, femora and ribs of extant squamates and semi-aquatic to fully marine tetrapods (Tables 2–4).

**Table 1:** List of the mosasauroid long bones analyzed in this study.

Taxon	Coll. Nb.	Age	Locality/Stratigraphy	Bone
*Clidastes* sp.	UCMP 34536		Niobrara Fm, Kansas USA	H
*Prognathodon kianda*	MGUAN-PA Unnb.	M	Latest Campanian, Bentiaba, Angola	LH
*Prognathodon* or *Mosasaurus*	MJP Unnb.	M	Latest Campanian, Bentiaba, Angola	H
*Prognathodon* or *Mosasaurus*	MGUAN-PA Unnb.	M	Latest Campanian, Bentiaba, Angola	RH
*Mosasaurus* sp.	MGUAN-PA Unnb.	M	Latest Campanian, Bentiaba, Angola	LH
*Prognathodon* sp.	IRScNB 1624	M	Ciply Phosphatic Chalk, Belgium	H*
Mosasaurinae indet.	SMU 76406	C	Ozan Fm, Texas, USA	H
Mosasaurinae indet.	SMU 76407	C	Ozan Fm, Texas, USA	H?
Mosasaurinae indet.	SMU 76407	C	Ozan Fm, Texas, USA	H?
*Dallasaurus turneri*	SMU 76386	T	Cedar Hill, Texas, USA	H*
	SMU 76529	T	Cedar Hill, Texas, USA	F* & Ri*
*Plotosaurus bennisoni*	UCMP 152664	M	Moreno Fm, California, USA	RRa
	UCMP 152554	M	Moreno Fm, California, USA	RRa
*Globidens* sp.	MGUAN-PA Unnb.	M	Latest Campanian, Bentiaba, Angola	LH
Mosasaurine indet.	MGUAN-PA Unnb.	M	Latest Campanian, Bentiaba, Angola	LH

* elements included in the LDA. Abbreviations- T: Turonian, S: Santonian, C: Campanian; M: Maastrichtian; L: left, R: right, H: humerus, Ra: radius, Ri: rib, T: tibia; Unnb: unnumbered. MGUAN-PA: Geological Museum, Universidade Agostinho Neto, Luanda, Angola (PaleoAngola collection).

**Table 2:** Non-mosasauroid humeri analyzed.

Systematic Position	Taxon	Abb.	Ecol.	Coll. Nb. & Reference
Ichthyosauria	*Stenopterygius* sp.	St	AS	Unnb, [20]
Placodontia	Placodont indet.	Pl	PAS	IGWH-9, [56]
Pachypleurosauria	*Anarosaurus* sp.	An	PAS	Wijk08–183, [56]
				Wijk09–58, [56]
Nothosauria	*Nothosaurus* sp.	No	PAS	IGWH-7, [56]
				IGWH-3, [56]
Cymatosauridae	*?Cymatosaurus* sp.	Cy	PAS	IGWH-6, [56]
Squamata	*Amblyrhynchus cristatus*	Am	ET	MZLUXX/6432
	*Tupinambis teguixin*	Tu	ET	MZLUXX/6410
	*Varanus bengalensis*	Vben	ET	MZLUXX/6393
	*Varanus candolineatus*	Vca	ET	MZLU896/3041
	*Varanus gouldi*	Vgo	ET	MZLU867/3039
	*Varanus indicus*	Vin	ET	MZLUXX/6390
	*Varanus niloticus*	Vni	ET	MZLU878/3026
Crocodilia	*Crocodylus* sp.	Cr	PAS	Unnb., [57]
Sirenia	*Trichechus manatus*	Tr	PAS	ZFMK 73.223
Pinnipedia	*Lutra lutra*	Lu	PAS	MNHN1906-236, [57]
	*Amblonyx cinereus*	Amb	PAS	MNHN277, [57]
	*Mirounga leonine*	Mi	AS	Unnb., [57]
Cetacea	*Delphinius delphis*	De	AS	MNHN AC 1880-1310
	*Phocoena phocoena*	Ph	AS	MNHN AC 1881-232
	*Tursiops truncatus*	Tur	AS	MNHN AC 1978-09

Abb: Abbreviations; lists of abbreviations used in Figure 5. Ecol: Ecological categories used in the LDA; ET: essentially terrestrial taxa; PAS: essentially or exclusively aquatic poorly active swimmers; AS: active swimmers. IGWH: Institute of Geosciences of the Martin-Luther-University Halle-Wittenberg, Germany; MZLU: Museum of Zoology, Lund University, Sweden; MNHN: Muséum National d’Histoire Naturelle, Paris, France; Wijk: Winterswijk collection in the National Museum of Natural History Naturalis, Leiden, The Netherlands; ZFMK: Zoologisches Forschungsmuseum Alexander Koenig, Bonn, Germany.

**Table 3:** Non-mosasauroid femora analyzed.

Systematic Position	Taxon	Abb.	Ecol.	Coll. Nb. & Reference
Placodontia	Placodont indet.	Pl	PAS	IGWH-23, [56]
Pachypleurosauria	*Anarosaurus* sp.	An	PAS	Wijk07–11, [56]
Nothosauria	*Nothosaurus* sp.	No	PAS	Wijk05–10, [56]
Cymatosauridae	*?Cymatosaurus* sp.	Cy	PAS	IGWH-24, [56]
				NME48000074, [56]
Squamata	*Amblyrhynchus cristatus*	Am	ET	MZLUXX/6432
	*Tupinambis teguixin*	Tu	ET	MZLUXX/6410
	*Varanus bengalensis*	Vben	ET	MZLUXX/6393
	*Varanus candolineatus*	Vco	ET	MZLU896/3041
	*Varanus gouldi*	Vgo	ET	MZLU867/3039
	*Varanus indicus*	Vin	ET	MZLUXX/6390
	*Varanus niloticus*	Vni	ET	MZLU878/3026
	*Dallasaurus turneri*	Da	ET	SMU 76386
Thalattosuchia	Teleosaurid indet.	Te	PAS	BHN 2R883, [58]
Crocodilia	*Alligator mississipiensis*	Al	PAS	SMNS 10481, [59]

Abbreviations are as in Table 2. BHN: Musée d’Histoire Naturelle de Boulogne-sur-Mer, France; NME: TwentseWelle, Enschede, The Netherlands; SMNS: Staatliches Museum für Naturkunde, Stuttgart, Germany.

**Table 4:** Comparative rib material analyzed.

Systematic Position	Taxon	Abb.	Ecol.	Coll. Nb. & Reference
Squamata	*Varanus rudicollis*	Vru	ET	MNHN AC 1983–6
	*Varanus varius*	Vva	ET	MNHN AC 1910–12
	*Carentonosaurus mineaui*	Ca	PAS	MNHN IMD 51
	*Dallasaurus turneri*	D		TMM 43209-1, [31]
	*Clidates* sp. (juvenile)	Cj	AS	UCMP 34536, [60]
	*Clidates* sp. (subadult)	Csa	AS	RMM 1287, [61]
	*Clidates* sp.	Ca	AS	RMM 1788, [31]
	*Tylosaurus* sp. (early juvenile)	Tej	AS	RMM 5610, [60]
	*Tylosaurus* sp. (late juvenile)	Tlj	AS	UW 1501.5, [60]
	*Platecarpus* sp. (late juvenile)	Plj	AS	UCMP 34781, [60]
	*Platecarpus* sp. (subadult)	Psa	AS	AMNH 1645, [61]
	*Platecarpus* sp.	Pa	AS	AMNH 1543, [11]
Thalattosuchia	Metriorhynchid indet.	Me	AS	MHBR 208, [58]
Crocodilia	*Crocodylus* sp.	Cr	PAS	Unnb., [62]
Pinnipedia	*Phoca vitulina*	Ph	AS	IPB M 60
	*Zalophus californianus*	Za	AS	ZFMK 49.98
	*Mirounga leonine*	Mi	AS	ZFMK 62.105
Cetacea	*Balaenoptera brydei*	Ba	AS	NSM M 32599

Abbreviations are as in Table 2. AMNHN: American Museum of Natural History, New York, USA; IPB: Institute for Paleontology, University of Bonn, Germany; MHBR: Muséum d’Histoire Naturelle du Havre, France; NSM: National Science Museum, Tokyo, Japan; RMM: former Red Mountain Museum, Birmingham, Alabama, USA (the collection is currently housed at the McWane Science Center, Birmingham, Alabama, USA); TMM: Texas Memorial Museum, Dallas, Texas, USA; UW: University of Wisconsin, Madison, Wisconsin, USA. The ontogenetic stage is indicated for non-adult specimens.

The specimens were loaned and permission for histological sampling was given by all institutions listed in Table 2.

Prior to sampling, the bones were measured, molded and casted. Approximately 5 mm thick blocks (transverse to the long axis) were removed from the mid-diaphyseal region of each element using a diamond saw, and then vacuum-embedded in a clear polyester resin to prevent shattering during slide preparation. Two thin sections approximately 50–100 µm thick were made from each block. The sections were observed under a LeicaH DM 2500 compound polarizing microscope equipped with a LeicaH DFC 420C digital camera. Additionally, the sections were scanned at high resolution (i.e., between 6400 and 12800 dpi) using an Epson V750-M Pro scanner, transformed into single-bit digital images using Photoshop CS3 (where black and white represent bone and cavities respectively), and analyzed using Bone Profiler [22].

Bone profile parameters for each section were measured or calculated following Laurin et al. [23]. These include: (1) C: compactness of the whole section; (2) P: the extent of the medullary cavity as measured by the relative distance from the center of the section to the point where the most abrupt change in compactness occurs; (3) S: the width of the transitional zone between the compact cortex and the medullary cavity as measured by the reciprocal of the slope of the compactness profile at the inflection point; (4) MD: maximum bone diameter at the level of section, which is considered as a proxy for body size; and (5) R/t: outside radius of the bone divided by the thickness of the cortex (cf. [24]).

In order to infer the lifestyle of the basal mosasaurine *Dallasaurus* we follow the methodology described by Germain and Laurin [25] by performing a linear discriminant analysis (LDA) to distinguish between: (1) essentially terrestrial; (2) essentially or exclusively aquatic poorly active swimmers; and (3) active swimmers. Prior to analysis, all data were transformed in order to meet assumptions of normality and homoscedasticicy: √P, logitS, logMD, and logR/t, except for compactness index (C), for which transformation was unnecessary (see Text S1). Moreover, the phylogenetic significance of the parameters was tested for all three categories of bones (i.e., humerus, femur and rib) on a consensus phylogeny (cf. [26]). Species mean values were used when several specimens (of comparable ontogenetic stage) were available for the same taxon. The descriptive K-statistic, which compares the observed phylogenetic signal in a trait (based only on the reference tree structure) with the signal under a Brownian motion model of trait evolution − following Blomberg et al. [27] − was provided. K values lower than 1 imply less similarity between relatives than expected under Brownian motion. Randomization tests were performed to test the phylogenetic signal of each parameter. Statistical analyses were performed using R Development Core Team [28].

The histological terminology is based primarily on Francillon-Vieillot et al. [29], and the systematics follow Bell and Polcyn [6].

## Results

### (a) Histological Features

#### Humerus

**Figure 1:**
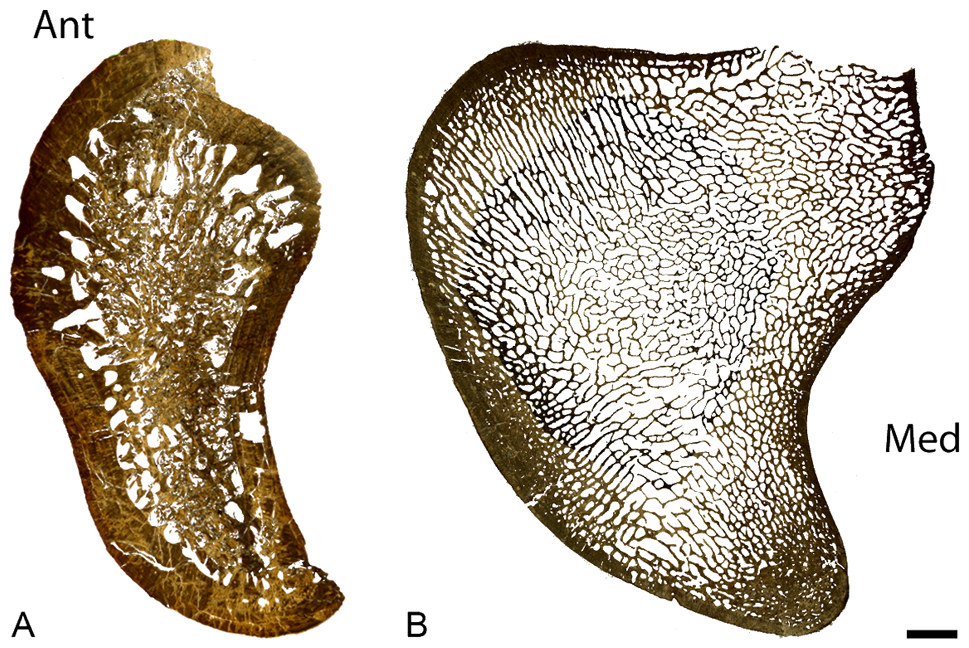
Humeral mid-diaphyseal sections. A, *Clidastes* sp. UCMP 34536. B, *Prognathodon* sp. IRScNB 1624. Ant: anterior, Med: medial. Scale bar equals A, 2 mm; B, 5.6 mm.

All sections, except that of *Dallasaurus* (see below), consist mainly of a spongiosa surrounded by a peripheral layer of compact cortical bone (Fig. 1). The cortical bone is rather thin in most taxa, but it is somewhat wider in *Clidastes*. Given the overall similarity in bone architecture between the different mosasaurine genera, we here provide a generalized description of humeri assigned to *Clidastes*, *Globidens*, *Mosasaurus*, and *Prognathodon*.

**Figure 2:**
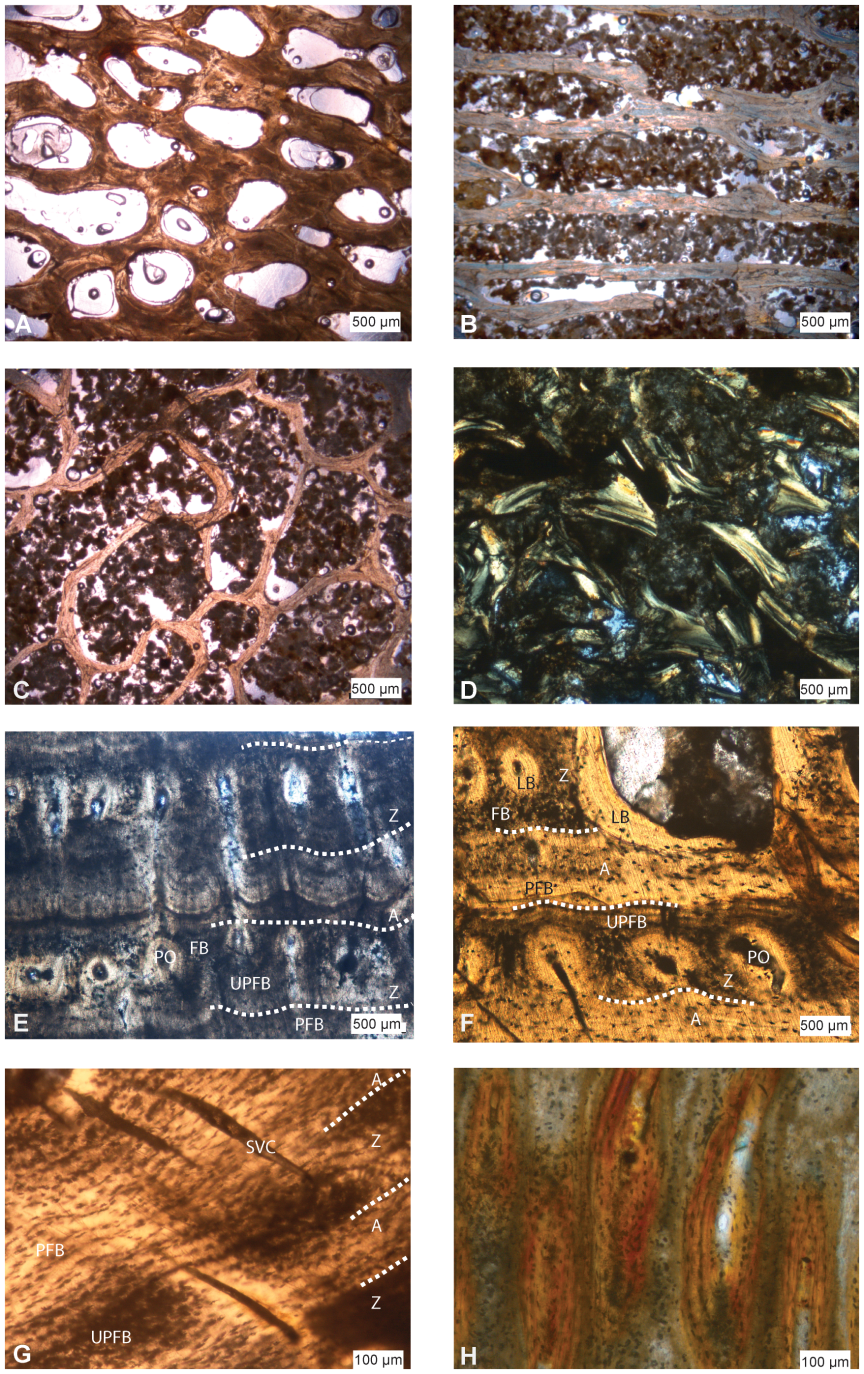
Microanatomical and histological features of mosasaurine humeri. A–C, *Prognathodon* sp., IRScNB 1624. Spongiosa with different tightness and organization of the trabecular network between the A, posterior, B, antero-lateral and C, inner regions. D–E, *Clidastes* sp., UCMP 34536. D, broken inner trabeculae displaying secondary lamellar bone (LB). E, cortex illustrating the different primary osseous tissues observable (parallel-fibered bone [PFB], unusual parallel-fibered bone [UPFB] and fibrous bone [FB]) in zones (Z) and annuli (A), and longitudinally oriented primary osteons (PO). F–G, Mosasaurinae indet., SMU 76406. F, as in E but also with clearly distinguishable lamellar bone (LB). G, cortex illustrating the change from PFB to UPFB between annuli and zones, and radially oriented simple vascular canals. H, Mosasaurinae indet., SMU 76407. Detail of osteons with extreme obliteration of the vascular spaces. SMU: Southern Methodist University, Shuler Museum of Paleontology, Dallas, Texas, USA.

**Figure 3:**
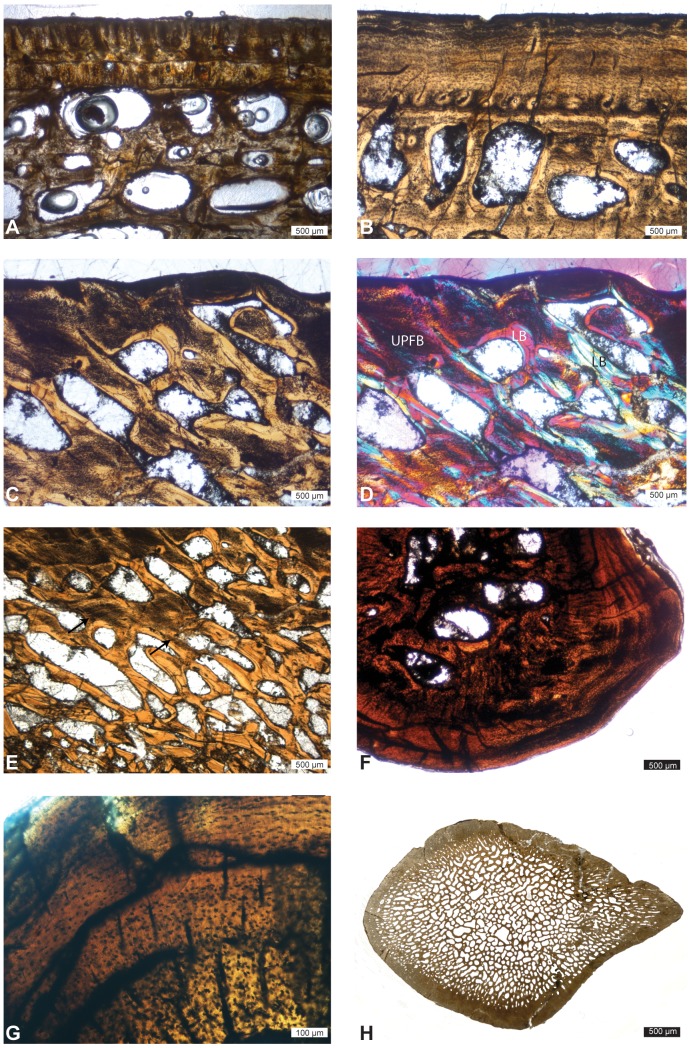
Histological and microanatomical features in mosasaurine long bones. A–G, Humeri. A, *Globidens* sp., PA Unnumbered. B-E, Mosasaurinae indet., SMU 76406. A–B, cortex showing the circumferential organization of the longitudinally oriented osteons and the increased resorption centripetally. C–D, external spongiosa in C, natural and D, polarized light (with gypsum filter) showing secondary deposits of parallel-fibered (PFB) and lamellar (LB) bone. E, transition from the external spongiosa (top) with important remains of primary (UPF) bone in the core of the trabeculae (as pointed by arrows), and inner spongiosa with exclusively secondary deposits of PFB and LB (bottom). F–G, *Dallasaurus turneri*, SMU 76386. F, part of the section illustrating the rather compact micro-organization. G, transition from the avascular external cortex made of PFB and the inner cortex made of UPFB and displaying radially oriented simple vascular canals. H, radius section of *Plotosaurus bennisoni*, UCMP 152664. Abbreviations as in Figure 2.

In most sections − except IRScNB 1624, here referred to *Prognathodon* sp. − the spongiosa is poorly preserved and most deep trabeculae are broken (Fig. 1A; IRScNB: Institut Royal des Sciences Naturelles de Belgique, Bruxelles, Belgium). However, the spongiosa is clearly heterogenous and various zones with distinct patterns occur (Fig. 1A). It is denser in the posterior part of the section, with reduced intertrabecular spaces (Figs. 2B–3A), whereas is it looser in the antero-lateral part, and much looser in the central part. This is in accordance with the observation that the central region is generally crushed during diagenesis (Fig. 1A). Antero-laterally, the trabeculae are oriented parallel to one another and they show a predominantly radial orientation (Figs. 2AB–3B); however, they appear more randomly oriented toward the central part of the section (Fig. 2C).

Four types of bone tissues are present in the cortex: (1) lamellar bone, with the lamellae displaying an alternate extinction in polarized light (Fig. 2D–F). The osteocyte lacunae are highly elongate and aligned parallel to the main direction of bone deposit; (2) a bone tissue with a mass birefringence in polarized light, which is characteristic of parallel-fibered bone. The cell lacunae are elongate and aligned parallel to the direction of bone deposit (Fig. 2G); (3) a tissue with mass birefringence under polarized light (a feature typical of parallel-fibered bone), but where large cell lacunae are present (a feature generally encountered only in fibrous bone; Fig. 2G). The osteocyte lacunae are either aligned parallel to one another (Fig. 2G) or more or less randomly arranged, which also indicates variations in growth speed; (4) Isotropic bone under polarized light with large and randomly shaped cell lacunae, corresponding to true fibrous bone (Fig. 2E–F). These four types of osseous tissue are hereafter referred to as: (1) lamellar bone (LB); (2) traditional parallel-fibered bone (PFB); (3) unusual parallel-fibered bone (UPFB); and (4) fibrous bone (FB).

In the cortex, LB is only rarely observed. It occurs in osteons and as secondary bone in areas of active remodelling (which are essentially restricted to the inner cortex). It is possible, especially in the medial and lateral parts of the sections, to observe distinct phases of growth through zones and annuli (Fig. 2E–G). Annuli are characterized by the unique occurrence of PFB, whereas zones exhibit UPFB, sometimes associated with local deposits of FB (Fig. 2E–F).

The vascularization consists of simple vascular canals and primary osteons oriented both longitudinally (Fig. 2E–F) and radially (Fig. 2G). Some oblique orientations also occur, as do a few anastomoses. The degree of vascularization and the orientation of the vascular canals are not homogeneous in any section. A predominantly longitudinal orientation is observed in the medial region, and only in the zones. The degree of vascularization is generally rather high. Substantial secondary endosteal deposits occur in the osteons, resulting in the quasi-obliteration of some vascular spaces (Fig. 2H). However, large cavities organized in concentric rows in the inner part of the cortex indicate strong resorption of the primary osteons (Fig. 3A–B). In the external spongiosa, the trabeculae often display some remains of primary bone (Fig. 3C–E), whereas they are completely remodelled in the deep spongiosa, where they instead consist of PFB and LB (Fig. 3E).

The humerus of *Dallasaurus* differs markedly from that of other hydropelvic mosasaurines. The osseous organization is not spongious; instead, the bone is relatively compact (Fig. 3F). A thick dense layer surrounds the medullary area, which contains a few large and randomly shaped intertrabecular spaces separated from one another by thick trabeculae. The periosteal bone consists in its periphery of PFB with aligned, elongate lacunae. Vascular canals are almost completely absent from this layer (Fig. 3F–G). Towards the medullary region, the osteocyte lacunae are larger and randomly oriented, as in UPFB, and simple, radially oriented vascular canals are present (Fig. 3G). Interestingly, locally in the periphery of the cortex, the cell lacunae are also larger, but elongated parallel to the direction of bone deposit. Remodelling has only occurred in the medullary region, where secondary deposits of parallel-fibered and lamellar bone are observed.

#### Other appendicular elements

The *Plotosaurus* radius sections show microanatomical (Fig. 2H) and histological features similar to those of the humeri described above. However, some differences are observed in the tightness of the spongiosa; it is tighter (i.e., more numerous trabeculae but smaller intertrabecular spaces) in UCMP 152554 than in UCMP 152664 (UCMP: University of California Museum of Paleontology, University of California, Berkeley, California, USA). This difference is consistent with the increase in number but decrease in the size of cavities with increased size of the individual, a condition also observed in extant squamates and hydropelvic mosasauroid vertebrae [12], [30].

**Figure 4:**
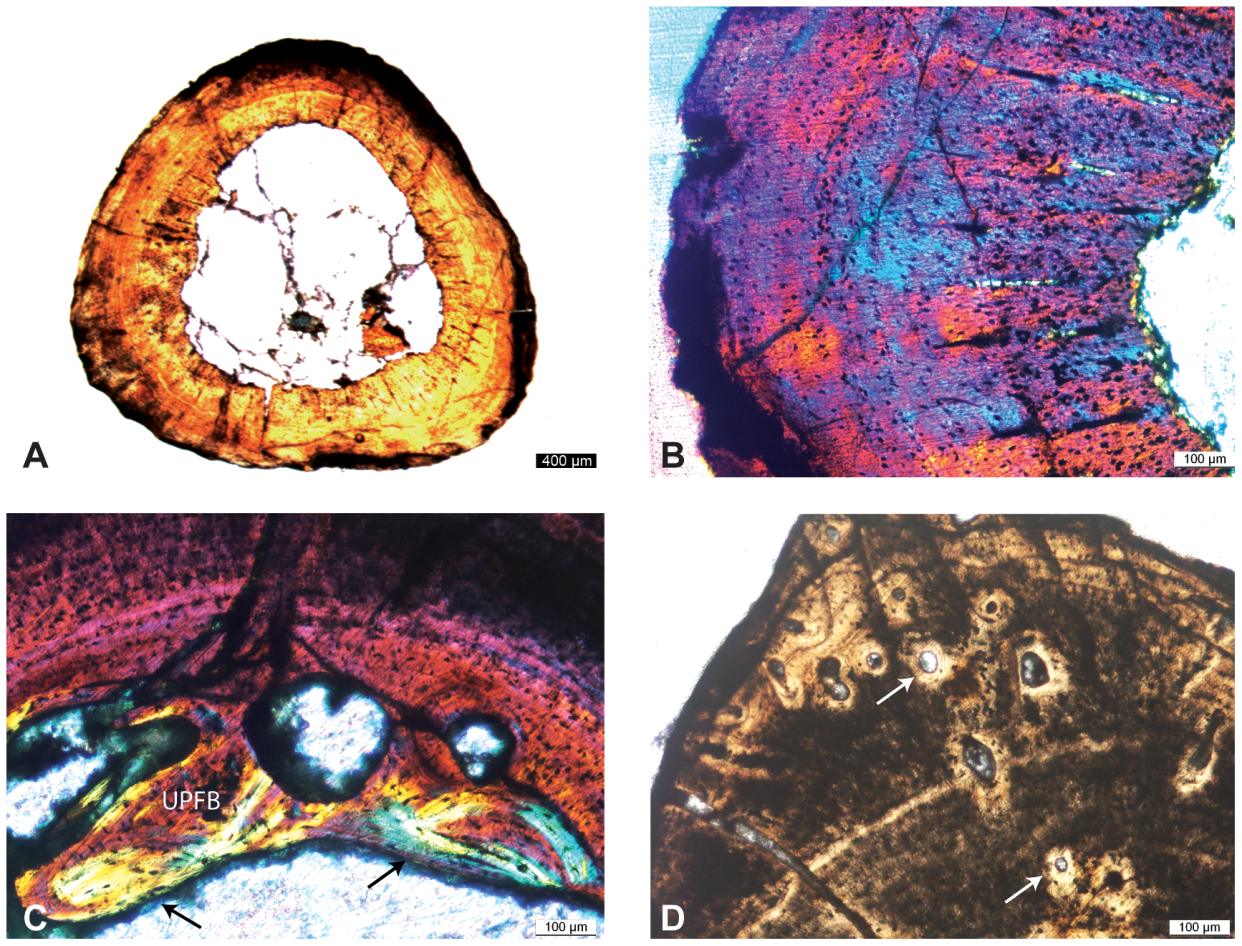
*Dallasaurus turneri*, SMU unnumbered specimen, microanatomical and histological features. A–B, femur. A, whole mid-diaphyseal section. B, transition from the avascular external cortex made of PFB to the inner cortex made of UPFB and displaying radially oriented simple vascular canals. C–D, rib. C, transitional area between the cortex and the medullary region showing some secondary LB and PFB as well as some remains of primary UPFB in the core of one of the remodelled trabeculae. D, longitudinally oriented primary osteons (indicated by arrows) in the cortex.

The femur and ribs of *Dallasaurus* show a tubular structure with a compact cortex and a large, open medullary cavity (Fig. 4A; [31]). The histological features of the femur are similar to those observed in the humerus (Fig. 4B), with the exception that secondary bone deposits are extremely rare. In the ribs, the cortical thickness is variable. The periosteal bone of the ribs consists essentially of PFB. A few deposits of UPBF are present locally in the periphery of the cortical bone, and a remnant of UPFB is located close to the medullary cavity. A few endosteal deposits of secondary PFB and LB occur in this area (Fig. 4C). The vascularization consists of only a few primary osteons oriented longitudinally, in that region of the bone where the cortex is the thickest (Fig. 4D).

### (b) Microanatomical Comparative Analysis

#### Humerus

**Figure 5:**
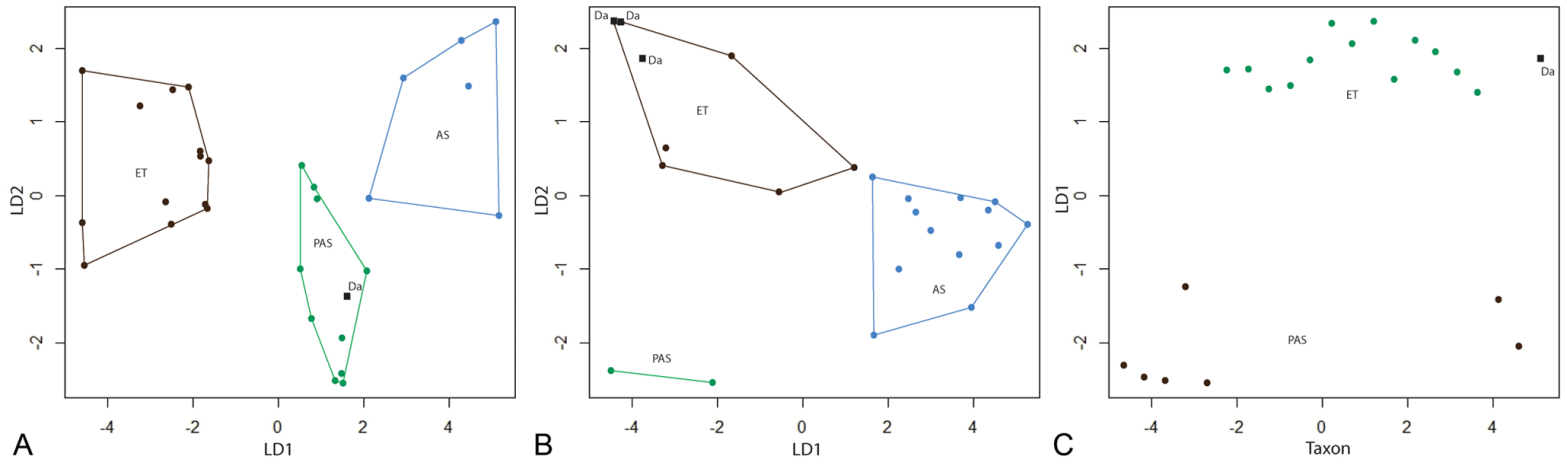
Results of the Linear Discriminant Analyses (LDA) performed on A, humeri, B, femora and C, ribs. LD1 and LD2: first and second discriminant axis, respectively. Polygons represent the boundaries of the ecological categories based on the comparative material (see Tables 2–4). Da, *Dallasaurus*.

The Linear Discriminant Analysis (LDA) for the humerus was very efficient: it correctly attributed the habitat for 97% (28 out of 29) of the taxa (100% of the “essentially terrestrial” [ET], 100% belonging to the “essentially or exclusively aquatic poorly active swimmers” [PAS] grouping, and 83% of the “active swimmers” [AS] grouping). The long bones of the ET taxa (i.e., all extant squamates) are characterized by a tubular structure with a layer of compact cortex surrounding an open medullary cavity. Variations in the relative proportions of the latter feature are observed between different taxa: for instance, *Varanus caudolineatus* has the smallest medullary cavity, whereas *V. indicus* and *V. bangalensis* have the widest. Most PAS display a relatively high compactness and a reduced medullary cavity with no clear contours in some taxa. Lutrines, in addition to one specimen of *Nothosaurus*, differ from this general trend with their subtubular and tubular structure (i.e., cortical “tube” surrounding the medullary area), respectively. Although this does not prevent these animals to group with the other PAS in the LDA, this difference should not be neglected. All AS (including *Mosasaurus*) are characterized by a spongious inner bone organization, generally surrounded by a thin layer of compact cortical bone. *Stenopterygius* (a “dolphin-like” ichthyosaur), which displays a much tighter spongiosa than do the other taxa, is the only fossil that does not group with the AS (but rather with the PAS) in the LDA analysis. The lifestyle of *Dallasaurus* was inferred as PAS (Fig. 5A), which is consistent with its highly compact inner bone structure and absence of a medullary cavity.

#### Femur

The LDA for the femur included only two ecological categories (ET and PAS). The two parameters S and P do not show a normal distribution for this sample. This is probably because the two categories have strongly distinct microanatomical features, as shown by the extreme efficiency of the LDA that correctly attributed the habitat for all taxa. ET taxa are characterized by a distinct tubular structure with a large medullary cavity, whereas the PAS taxa display a much higher compactness and a reduced medullary cavity (which is sometimes surrounded by a spongiosa). The lifestyle of *Dallasaurus* was inferred as similar to those of ET taxa (Fig. 5B).

#### Ribs

The LDA was also efficient for the ribs. The habitat was correctly attributed for 90% (18 out of 20) of the taxa (80% of the ET, 100% of the PAS and 92% of the AS). The ribs of ET (extant squamates) display a typical tubular structure. The PAS show a very compact structure with a much reduced medullary cavity. AS are generally characterized by a relatively lower compactness of their bones and a spongious inner organization. Presumably as a result of its low tightness (i.e., few cavities and thick trabeculae), the *Clidastes* adult section was erroneously attributed to the PAS grouping. The *V*. *varius* section does not show a tubular structure but instead several cavities are present in the periphery of the medullary cavity. This probably explains why the LDA attributed it to the AS grouping. The lifestyle of *Dallasaurus*, as suggested by its distinct tubular rib organization, was inferred as that of the ET (Fig. 5C).

#### Phylogenetic signal

The K values for all bones are lower than 1. For the humerus, they vary between 0.57 and 0.89. However, the randomization tests indicate a significant phylogenetic signal for all parameters. For the femur, K values vary between 0.41 and 0.77. The randomization tests indicate a significant phylogenetic signal for C, R/t and P. As for the ribs, K values vary between 0.38 and 0.49 and the randomization tests indicate an absence of any significant phylogenetic signal for all parameters. This discrepancy strongly suggests that, if some parameters do display a phylogenetic signal, it should be rather weak. Moreover, it is probably linked to the small size and phylogenetic diversity of our sample.

## Discussion

### (a) Adaptive and Evolutionary Implications

During the early Late Cretaceous, mosasauroids radiated rapidly in the marine realm [32]. Over a few million years, they became larger, more diverse, adapted to an open-marine life, and attained an intercontinental distribution [1], [32], [33]. Members of the Russellosaurina were the first ones to do so, and by the Middle Turonian (∼92 Ma) the largest taxa reached body lengths of about six metres [34]. In contrast to russellosaurines, the best known mid-Turonian mosasaurine, *Dallasaurus*, was small and had a monitor lizard-like body with plesiopedal limbs [6]. Subsequent mosasaurines during the Late Turonian to Santonian interval showed modest increases in body size and remained endemic to the Gulf margins and Western Interior Seaway of North America [34]. Only later in the Campanian did mosasaurines disperse geographically and eventually become truly colossal in size (i.e., 10–15 meters in overall body length) [3], [35].

**Figure 6:**
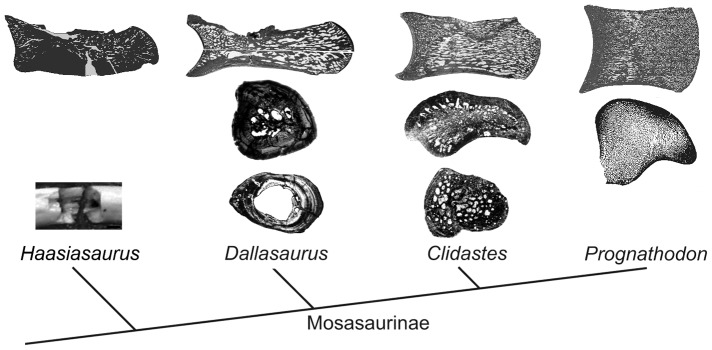
Evolution of microanatomical features in mosasaurine (from top to bottom) vertebrae, humeri and ribs.

The transition of mosasauroids from land-dwelling forms to highly derived marine-adapted forms is not only manifested at the gross anatomic level (e.g., [4], [5], [19], [36]), but is also readily apparent in the microanatomical and histological features of their skeleton, which undergo profound modifications. For instance, plesiopedal and plesiopelvic mosasauroids display bone mass increase (BMI) in at least their dorsal vertebrae and ribs [10], which suggests that they utilized hydrostatic buoyancy and body trim control when submerged. From these presumably poorly active swimmers distinct patterns of mosasaurine evolution illustrate trends toward more energy-efficient locomotion and optimization for a pelagic lifestyle, including: (1) the development of a streamlined body powered by a two-lobed tail fin [4], [5]; (2) the exploitation of increasingly offshore foraging habitats [37]; and (3) the worldwide distribution during the Campanian-Maastrichtian interval [1], [33]. In active swimmers, buoyancy and body trim control is ensured hydrodynamically [38]. The transition from a hydrostatic to a hydrodynamic buoyancy and body trim control is evidenced as profound changes in the microanatomy of mosasaurine axial and appendicular bones (Fig. 6); a shift in microanatomical specializations that is comparable to that observed in early Cetacea (cf. [39], [40]). A shift from bone mass increase (BMI) (as in some archaeocetes) to a spongious osseous organization (as in all neocetes) is known to have occurred early in cetacean evolution (e.g. [39]), and presumably reflects a similar shift in ecology, from poorly active swimmers in shallow waters to active pelagic foragers in open-sea environments.

### (b) Mosasaurine Growth Rates and Basal Metabolic Rates

Parallel-fibered bone (PFB) has previously been recorded in the cortex of vertebrae belonging to hydropelvic mosasauroids [12], [17]. PFB is also the dominant type of periosteal tissue found in the pro- and epipodials of mosasaurines; however, even though the organization of the collagenous wave is similar to that of typical PFB, the osteocyte lacunae are unusually large, irregularly shaped and randomly oriented. A relatively poor degree of osseous organization is generally attributed to elevated growth rates [41], [42], and is thus normally encountered only in fibrous bone (FB). The peculiar sort of PFB found in mosasaurine long bones, herein referred to as unusual parallel-fibered bone (UPFB), may correspond to a tissue described by Ricqlès et al. ([43], p. 75) as illustrating a “modification of the fibro-lamellar complex”, whose “cortex appears lamellar under polarized light at low magnification, because this kind of tissue is not as woven as in typical dinosaurian fibro-lamellar bone tissues”. The occurrence of UPFB probably reflects a growth rate intermediate between that of typical PFB and FB. A reinvestigation of the vertebral sections described by Houssaye and Bardet [12] revealed local occurrences of UPFB, but to a much lesser extent than in long bones (A.H. pers. obs.). These observations suggest that, at least during late ontogeny (the record of earlier ontogenetic stages is lost to bone resorption) the long bones of mosasaurines grew faster in width than did the vertebrae. Because bone shape is maintained during ontogeny, this suggestion can be generalized to the whole bone growth. This is in agreement with a previous observation [44] that the greatest allometric growth occurs within the humerus in *Clidastes*. A positive allometry of the humerus when compared to vertebrae and/or overall body size was also noticed in the basal ichthyosaur *Chensaurus chaoxianensis* (Grippidae; [45]).

The orientation of the vascular network, which consists of both primary osteons and simple vascular canals, varies within a section, from predominately radial to longitudinal. A comparable variation was also observed in the vertebrae examined by Houssaye and Bardet [12], and probably expresses local differences in growth rate.

Mosasaur growth rates have been interpreted to be considerably higher than are those in extant squamates [12], which display true PFB, where simple, radially oriented vascular canals only occur in skeletal elements of large-sized taxa [46]. Within Mosasauroidea, the growth rates of plesiopelvic forms were considered to be higher than were those of hydropelvic ones by Houssaye and Bardet [12], based on the exclusively radial orientation of the vascular network in vertebrae of the former morphotype (compared to the primarily longitudinal orientation of those in the latter). However, it has also been suggested [17] that hydropelvic mosasauroids might have had growth rates similar to those of plesiopelvic forms at early ontogenetic stages, and that the rates then decreased during protracted growth. The long bone histology of plesiopelvic mosasauroids has yet to be examined, in part due to the rarity of specimens and access to material that can be subjected to destructive analysis; thus, at this time no comparisons can be made with these most primitive mosasauroids.

Bone growth rate is indirectly linked to basal metabolic rate [47], [48], [49]. Thus, although with caution, bone tissue can be used to make inferences about thermal physiology and basal metabolic rate [50]. The dominance of fibro-lamellar bone (i.e., fibrous periosteal bone with lamellar bone in the osteons) in ichthyosaurs and plesiosaurs (see review in [13]) contrasts with the largely parallel-fibered bone found in hydropelvic mosasauroids (of comparable ontogenetic stage), and probably reflects higher growth rates in the former two groups (which have previously been considered to have had rather high basal metabolic rates [51], [52]). Bernard et al. [53] suggested that hydropelvic mosasauroids might have been partially homeothermic based on the supposedly high body temperatures (between 35±2°C and 39±2°C) they estimated from dental isotopic data. Motani [54] indicated that a bias may exist in Bernard et al.’s [53] results (arising from time dependent depletion of δ^18^O), and hence lowered these values and suggested that hydropelvic mosasauroids might have been gigantothermic, i.e., able to maintain elevated body temperatures by virtue of large body size and possibly insulation [55]. The predominance of parallel-fibered bone in mosasauroids, as in the modern leatherback turtle *Dermochelys*, supports this conclusion [13]; however, the occurrence of UPFB suggests that hydropelvic mosasauroid basal metabolic rates were higher than that of *Dermochelys*, although lower than those of plesiosaurs and ichthyosaurs, supporting the conclusions of Bernard et al. [53].

### (c) *Dallasaurus* – a Peculiar Basal Mosasaurine

Whereas all hydropedal mosasaurines display a spongious inner bone architecture characteristic of efficient swimmers, *Dallasaurus* displays tubular ribs and femora in similarity with extant terrestrial and semi-aquatic squamates; yet its humeri are osteosclerotic. Bone mass increase (BMI; including osteosclerosis) is often present in bottom walkers and poorly efficient swimmers that move slowly at shallow water depths. However, BMI is generally concentrated in the antero- and mid-dorsal regions of the body, where it counterbalances the lightness of the air-filled lungs. This is not the case in *Dallasaurus*, whose vertebrae show a microanatomical organization similar to that of other hydropelvic mosasauroids; i.e., a largely spongious organization (cf. [12]), and the ribs are tubular with a wide medullary cavity. The occurrence of BMI merely in the forelimbs has so far not been encountered in any other aquatic amniote (cf. [8]).

*Dallasaurus* possesses UPFB within the shaft of its long bones, although this osseous tissue is essentially restricted to the inner part of the cortex, where simple, radially oriented vascular canals occur in abundance. Conversely, most of the outer cortex consists of avascular, true PFB. The transition in both organization and vascularization indicates a decrease in growth rate during ontogeny. Despite a relatively limited amount of remodelling, no zonality seems to be present in the propodials, a feature otherwise frequently found in long bones of marine reptiles [13]. However, the dissimilarity in bone microarchitecture between the inner and outer cortex might not only reflect a relative decrease in growth speed, but may also represent a growth cycle; i.e., a zone (when growth is active) and an annulus (when growth speed decreases). This conclusion is corroborated by the local presence of UPFB in the periphery of the cortex, which could represent the beginning of a second growth cycle, and which suggests that the organism was still in an active growth phase at the time of death. If this is true, then the two specimens analyzed herein would have died within their second year. A juvenile ontogenetic stage for a third specimen of *Dallasaurus* was also suggested by the occurrence of significant remains of calcified cartilage far from the articular surfaces in one vertebral centrum (A.H. pers. obs.; cf. [12]). This is intriguing, because it would imply that *Dallasaurus* is currently only known from juvenile (and similar-sized) individuals. An analysis of the distribution of various anatomical characters within both juvenile and adult mosasauroids is required in order to determine which states could diagnose a juvenile ontogenetic stage; the high degree of paedomorphosis observed in mosasauroids [31] should naturally been taken into consideration.

*Dallasaurus* clearly differs from the other hydropelvic mosasauroids in its microanatomical features. Previous studies have shown that, within mosasauroids at least, bone microanatomical specializations do not vary during ontogeny [17]. Hence, the peculiar inner bone organization seen in *Dallasaurus* could not be attributed to a potential juvenile ontogenetic stage. *Dallasaurus* highlights a third ecological “grade” within mosasaurines (and mosasauroids) with spongious vertebrae, tubular ribs and femora, and osteosclerotic humeri, suggesting a more progressive ecological transition than previously hypothesised.

## Conclusions

1. Our analysis of hydropelvic mosasaurine long bones showed that the dominant type of periosteal bone tissue is parallel-fibered. Moreover, our investigation revealed the presence of a peculiar type of osseous tissue (herein referred to as unusual parallel-fibered bone), which probably reflects a growth rate intermediate between that of parallel-fibered and fibrous bone. Its occurrence in hydropelvic mosasaurines suggests that their basal metabolic rates were intermediate between that of the extant leatherback turtle *Dermochelys* and those inferred for plesiosaurs and ichthyosaurs.
2. *Dallasaurus* microanatomical features differ from those of other mosasaurines. With its spongious vertebrae, tubular ribs and femora, and osteosclerotic humeri, *Dallasaurus* highlights an intermediate evolutionary stage among mosasauroids, between plesiopelvic and hydropedal forms. This suggests that mosasauroid microanatomical adaptations to an obligate open-marine life were more gradual than previously thought. The heterogeneity of the microanatomical features in *Dallasaurus*’s skeleton also highlights the importance of analyzing bones from multiple anatomical regions.
3. The more complete image of the various microanatomical trends observed in mosasaurine mosasauroids strongly supports the evolutionary convergence between this squamate lineage and cetaceans in the ecological transition from a coastal to a pelagic lifestyle, and suggests a comparable underlying mechanism of skeletal adaptation to a an open-marine lifestyle.

## Supporting Information

Text S1 Tables presenting the values gathered for the different microanatomical parameters used for in Linear Discriminant Analyses.
(DOC)
