# Supplementary material for: Microanatomical and Histological Features in the Long Bones of Mosasaurine Mosasaurs (Reptilia, Squamata) – Implications for Aquatic Adaptation and Growth Rates
Source: PLoS One. 2013 Oct 16;8(10):e76741. doi: 10.1371/journal.pone.0076741 (PMC3797777; doi:10.1371/journal.pone.0076741)
Supplement: Text S1 — Tables presenting the values gathered for the different microanatomical parameters used for in Linear Discriminant Analyses. (DOC) [file pone.0076741.s001.doc]

**Text S1.** Tables presenting the values obtained for the different microanatomical parameters used for the Linear Discriminant Analyses.

**Humeri**

| Systematic Position | Taxon | Coll. Nb. | MD  (mm) | C | R/t | S | P |
| --- | --- | --- | --- | --- | --- | --- | --- |
| Ichthyosauria | *Stenopterygius* sp. | Unnb. | 18.7 | 0.727 | 2.3390 | 0.0483124 | 0.5724645 |
| Placodontia | Placodont indet. | IGWH-9 | 14.2 | 0.899 | 1.1559 | 0.1139515 | 0.1349010 |
| Pachypleurosauria | *Anarosaurus* sp. | Wijk08-183 | 6.9 | 0.895 | 1.4324 | 0.0549574 | 0.3018499 |
|  |  | Wijk09-58 | 7.7 | 0.884 | 1.2055 | 0.1397245 | 0.1704652 |
| Nothosauria | *Nothosaurus* sp. | IGWH-7 | 15.1 | 0.650 | 2.4039 | 0.0345037 | 0.5840030 |
|  |  | IGWH-3 | 10.3 | 0.932 | 1.1885 | 0.1057664 | 0.1585959 |
| Cymatosauridae | *?Cymatosaurus* sp. | IGWH-6 | 14.1 | 0.914 | 1.3297 | 0.0667903 | 0.2479682 |
| Squamata | *Amblyrhynchus cristatus* | LXX/6432 | 3.7 | 0.668 | 2.5241 | 0.0157934 | 0.6038260 |
|  |  |  |  | 0.647 | 2.4345 | 0.0210183 | 0.5892304 |
|  | *Tupinambis teguixin* | LXX/6410 | 4.1 | 0.754 | 1.9668 | 0.0181038 | 0.4915549 |
|  |  |  |  | 0.745 | 2.0004 | 0.0179017 | 0.5001072 |
|  | *Varanus bengalensis* | LXX/6393 | 1.2 | 0.456 | 3.7437 | 0.0321171 | 0.7328873 |
|  |  |  |  | 0.470 | 3.6001 | 0.0267363 | 0.7222287 |
|  | *Varanus candolineatus* | L896/3041 | 0.9 | 0.799 | 1.8033 | 0.0077829 | 0.4454601 |
|  |  |  |  | 0.870 | 1.5568 | 0.0081731 | 0.3576463 |
|  | *Varanus gouldi* | L867/3039 | 3.8 | 0.634 | 2.5021 | 0.0191161 | 0.6003418 |
|  | *Varanus indicus* | LXX/6390 | 1.1 | 0.484 | 3.4906 | 0.0093432 | 0.7135184 |
|  |  |  |  | 0.524 | 3.1738 | 0.0181038 | 0.6849221 |
|  | *Varanus niloticus* | L878/3026 | 2.2 | 0.741 | 2.0184 | 0.0147227 | 0.5045492 |
|  |  |  |  | 0.784 | 1.8532 | 0.0151942 | 0.4604073 |
|  | *Dallasaurus turneri* | SMU Unnb. | 4.1 | 0.898 | 1.7329 | 0.0586974 | 0.4229333 |
|  | *Mosasaurus* sp. | IRSNB 1624 | 74.9 | 0.594 | 1.9705 | 0.2880912 | 0.4925086 |
| Crocodilia | *Crocodylus* sp. | Unnb. | 13.1 | 0.822 | 1.5865 | 0.0401926 | 0.3696864 |
| Sirenia | *Trichechus manatus* | ZFMK 73.223 | 30.2 | 0.847 | 1.6456 | 0.0479158 | 0.3923028 |
| Pinnipedia | *Lutra lutra* | MNHN 1906-236 | 12.0 | 0.699 | 2.1403 | 0.0450924 | 0.5327749 |
|  | *Amblonyx cinereus* | MNHN 277 | 5.4 | 0.652 | 2.3996 | 0.0610522 | 0.5832700 |
|  | *Mirounga leonine* | Unnb. | 72.4 | 0.349 | 7.6633 | 0.1039685 | 0.8695086 |
| Cetacea | *Delphinius delphis* | MNHN AC 1880-1310 | 26.0 | 0.348 | 3.7579 | 0.1679249 | 0.7338938 |
|  | *Phocoena phocoena* | MNHN AC 1881-232 | 19.8 | 0.424 | 8.3139 | 0.0734913 | 0.8797193 |
|  | *Tursiops truncatus* | MNHN AC 1978-09 | 40.9 | 0.464 | 4.7064 | 0.1143333 | 0.7875239 |

**Femora**

| Systematic Position | Taxon | Coll. Nb. | MD  (mm) | C | R/t | S | P |
| --- | --- | --- | --- | --- | --- | --- | --- |
| Ichthyosauria | *Stenopterygius* sp. | Unnb. | 16.0 | 0.7 | 2.8709 | 0.0134536 | 0.6516773 |
| Placodontia | Placodont indet. | IGWH-23 | 19.1 | 0.8 | 1.4311 | 0.1885487 | 0.3012407 |
| Pachypleurosauria | *Anarosaurus* sp. | Wijk07-11 | 4.1 | 0.9 | 1.3314 | 0.0222741 | 0.2489056 |
| Nothosauria | *Nothosaurus* sp. | Wijk05-10 | 8.9 | 0.9 | 1.3545 | 0.0969048 | 0.2617038 |
| Cymatosauridae | *?Cymatosaurus* sp. | IGWH-24 | 9.1 | 0.9 | 1.4927 | 0.0118949 | 0.3300531 |
|  |  | NME48000074 | 6.8 | 1.0 | 1.3232 | 0.0115092 | 0.2442603 |
| Squamata | *Amblyrhynchus cristatus* | LXX/6432 | 3.9 | 0.6 | 2.6624 | 0.0139648 | 0.6243988 |
|  |  |  |  | 0.6 | 2.6325 | 0.0172845 | 0.6201386 |
|  | *Tupinambis teguixin* | LXX/6410 | 5.2 | 0.7 | 2.3102 | 0.016031 | 0.5671406 |
|  |  |  |  | 0.6 | 2.4347 | 0.0130202 | 0.5892754 |
|  | *Varanus bengalensis* | LXX/6393 | 1.0 | 0.4 | 4.3155 | 0.0159133 | 0.7682758 |
|  |  |  |  | 0.5 | 3.0458 | 0.0106288 | 0.6716775 |
|  | *Varanus candolineatus* | L896/3041 | 0.9 | 0.7 | 2.0527 | 0.011938 | 0.5128339 |
|  |  |  |  | 0.6 | 2.4800 | 0.0071805 | 0.5967695 |
|  | *Varanus gouldi* | L867/3039 | 4.8 | 0.6 | 2.8709 | 0.0134536 | 0.6516773 |
|  | *Varanus indicus* | LXX/6390 | 1.1 | 0.5 | 3.6492 | 0.0160163 | 0.7259656 |
|  |  |  |  | 0.4 | 3.9692 | 0.0157896 | 0.7480584 |
|  | *Varanus niloticus* | L878/3026 | 3.0 | 0.7 | 2.2352 | 0.0152114 | 0.5526032 |
|  |  |  |  | 0.7 | 2.0494 | 0.0089623 | 0.5120601 |
|  | *Dallasaurus turneri* | SMU Unnb. | 3.2 | 0.7 | 2.4482 | 0.0222476 | 0.5915354 |
| Thalattosuchia | *Teleosaurid* sp. | BHN 2R883 | 34.6 | 0.8 | 1.5748 | 0.0697118 | 0.3649867 |
| Crocodilia | *Alligator mississipiensis* | SMNS 10481 | 17.4 | 0.9 | 1.426 | 0.0741995 | 0.2987297 |

**Ribs**

| Systematic Position | Taxon | Coll. Nb. | MD  (mm) | C | R/t | S | P |
| --- | --- | --- | --- | --- | --- | --- | --- |
| Squamata | *Eublepharus macularius* | MNHN SQ-Vert 3 | 1.1 | 0.785 | 1.8434 | 0.0300651 | 0.4575179 |
|  | *Varanus rudicollis* | MNHN AC 1983-6 | 4.1 | 0.886 | 1.4784 | 0.0369540 | 0.3236085 |
|  | *Varanus varius* | MNHN AC 1910-12 | 4.8 | 0.803 | 1.7288 | 0.0705223 | 0.4215797 |
|  | *Carentonosaurus mineaui* | MNHN IMD 51 | 2.7 | 0.989 | 1.0653 | 0.0366667 | 0.0612749 |
|  | *Dallasaurus turneri* | SMU Unnb. | 3.2 | 0.736 | 2.0265 | 0.0351648 | 0.5065484 |
|  |  |  |  | 0.731 | 2.0420 | 0.0390283 | 0.5102844 |
|  |  | TMM 43209 | 5.5 | 0.753 | 1.9430 | 0.0384333 | 0.4853297 |
|  | *Clidates* sp. | UCB 34536 | 8.3 | 0.811 | 2.3094 | 0.0574925 | 0.5669867 |
|  | *Clidates* sp. | RMM 1287 | 6.4 | 0.792 | 1.7795 | 0.0708444 | 0.4380413 |
|  | *Clidates* sp. | RMM 1788 | 8.1 | 0.827 | 1.1881 | 0.2837631 | 0.1582990 |
|  | *Tylosaurus* sp. | RMM 5610 | 12.5 | 0.809 | 1.7509 | 0.1025255 | 0.4288636 |
|  | *Tylosaurus* sp. | UW 1501.5 | 15.8 | 0.658 | 1.9853 | 0.2098731 | 0.4962931 |
|  | *Tylosaurus* sp. | RMM 1913 | 21.3 | 0.566 | 16.2849 | 0.0248634 | 0.9385933 |
|  | *Platecarpus* sp. | UCB 34781 | 12.0 | 0.85 | 2.6830 | 0.0698598 | 0.6272811 |
|  | *Platecarpus* sp. | AMNH 1645 | 20.3 | 0.814 | 1.4766 | 0.0812496 | 0.3227797 |
|  | *Platecarpus* sp. | AMNH 1543 | 16.1 | 0.667 | 2.5687 | 0.2147486 | 0.6107024 |
|  | *Epicrates cenchria* | ZFMK 86470 | 1.3 | 0.925 | 1.3671 | 0.0211626 | 0.2685458 |
|  |  |  | 1.3 | 0.939 | 1.3202 | 0.0239641 | 0.2425327 |
| Thalattosuchia | Metriorhynchid indet. | MHBR 208 1969 | 16.9 | 0.683 | 2.1050 | 0.1122048 | 0.5249316 |
| Crocodilia | *Crocodylus* sp. | Unnb. | 4.5 | 0.974 | 1.0946 | 0.0800921 | 0.0864575 |
| Pinnipedia | *Phoca vitulina* | IPB M 60 | 7.8 | 0.436 | 5.8281 | 0.0986008 | 0.8284171 |
|  | *Zalophus californianus* | ZFMK 49.98 | 13.7 | 0.505 | 4.1028 | 0.1560724 | 0.7562651 |
|  | *Mirounga leonine* | ZFMK 62.105 | 25.3 | 0.361 | 7.4667 | 0.1216663 | 0.8660715 |
| Cetacea | *Balaenoptera brydei* | NSM M 32599 | 45.8 | 0.611 | 3.0653 | 0.0742023 | 0.6737630 |
